# HER2 expression status in diverse cancers: review of results from 37,992 patients

**DOI:** 10.1007/s10555-015-9552-6

**Published:** 2015-02-25

**Authors:** Min Yan, Maria Schwaederle, David Arguello, Sherri Z. Millis, Zoran Gatalica, Razelle Kurzrock

**Affiliations:** 1 Center for Personalized Cancer Therapy, UC San Diego Moores Cancer Center, 3855 Health Sciences Drive, MC #0658, La Jolla, CA 92093-0658 USA; 2 Caris Life Sciences, Phoenix, AZ USA

**Keywords:** HER2 overexpression, Cancer, IHC, FISH, HER2 amplification

## Abstract

Human epidermal growth factor receptor 2 (HER2) amplification/overexpression is an effective therapeutic target in breast and gastric cancer. Although HER2 positivity has been reported in other malignancies, previous studies generally focused on one cancer type, making it challenging to compare HER2 positivity across studies/malignancies. Herein, we examined 37,992 patient samples for HER2 expression (+/− amplification) in a single laboratory. All 37,992 patients were tested by immunohistochemistry (IHC); 21,642 of them were also examined for HER2 amplification with either fluorescent *in situ* hybridization (FISH) (11,670 patients) or chromogenic *in situ* hybridization (CISH) (9,972 patients); 18,262 patients had tumors other than breast or gastric cancer. All tissues were analyzed in a Clinical Laboratory Improvement Amendments (CLIA) laboratory (Caris Life Sciences) at the request of referring physicians. HER2 protein overexpression was found in 2.7 % of samples. Over-expressed HER2 was detected predominantly in malignancies of epithelial origin; for cancers derived from mesenchyme, neuroendocrine tissue, central nervous system, and kidney, HER2 expression and amplification were remarkably rare or non-existent. Bladder carcinomas, gallbladder, extrahepatic cholangiocarcinomas, cervical, uterine, and testicular cancers showed HER2 positivity rates of 12.4, 9.8, 6.3, 3.9, 3.0, and 2.4 %, respectively. HER2 overexpression and/or amplification is frequently found across tumor types. These observations may have significant therapeutic implications in cancers not traditionally thought to benefit from anti-HER2 therapies.

**Electronic supplementary material:**

The online version of this article (doi:10.1007/s10555-015-9552-6) contains supplementary material, which is available to authorized users.

## Introduction

The oncogenic potential of human epidermal growth factor receptor 2 (HER2) has been firmly established in preclinical and clinical settings. Among all four HER family proteins, HER2 has the strongest catalytic kinase activity and functions as the most active signaling complex of the HER family after dimerization with other HER family members [1, 2]. Overexpression of HER2 in breast cancer leads to increased homodimerization (HER2:HER2) and heterodimerization (e.g., HER2:HER3), which initiates a strong pro-tumorigenic signaling cascade [3]. Overexpression of HER2 protein drives malignant transformation in cell culture and transgenic mouse models [4, 5]. The anti-HER2 antibody trastuzumab represents an effective, targeted therapy with significant efficacy in treatment of HER2-positive breast and gastric cancer [6, 7]. Indeed, trastuzumab in combination with cisplatin and a fluoropyrimidine (capecitabine or 5-fluorouracil) has been approved for the treatment of patients with HER2 overexpressing metastatic gastric or gastroesophageal (GE) junction adenocarcinoma, who have not received prior treatment for metastatic disease [8]. The latter approval is based on a significant improvement in median overall survival (OS) of 2.5 months with trastuzumab plus chemotherapy treatment compared to chemotherapy alone, demonstrated in an international, multicenter, open-label, randomized clinical trial, BO18255 (ToGA trial) [6]. Furthermore, the family of approved anti-HER2 agents has been expanding in recent years, with the addition of small molecule inhibitors (e.g., lapatinib), antibodies (pertuzumab), and an antibody-drug conjugate (ado-trastuzumab emtansine, T-DM1). Used alone or in combination with other targeting agents or chemotherapy, these anti-HER2 agents have remarkably improved the outcome of patients with HER2-positive breast cancer [9, 10].

Breast and gastric cancers cases demonstrate a substantial HER2 protein overexpression by immunohistochemistry, predominantly driven by *HER2* amplification at the DNA level. The majority of studies of HER2-targeting therapies have been focused on this group of HER2-positive breast or gastric cancer cases. However, HER2 amplification/overexpression is known to exist in a non-negligible subset of cancers outside of breast and stomach. For example, approximately 15–37 % of salivary duct carcinomas exhibit 3+ HER2 expression [11, 12]. Other malignancies including, but not limited to, non-small cell lung (NSCLC), ovarian, colon, and pancreatic cancer, overexpress HER2 protein and/or exhibit gene amplification in a variable percentage of cases [13, 14]. Additionally, mutations in *HER2* have been described in a small subset of cancers of the breast, lung, ovary, and colon [13]. Anecdotal reports of patients with diverse cancers and *HER2* amplification or mutation responding to anti-HER2 agents have been published [15–18], implying a potential role for anti-HER2 agents outside of breast and gastric cancer.

Multiple reports of studies evaluating the percentage of HER2 positivity in individual cancer types have shed some light on the distribution of HER2 positivity across individual cancer types [13]; however, given a lack of standardized methodology and interpretation criteria, it is challenging to compare the rate of HER2 positivity across studies and tumor types. Herein, we reviewed 37,992 patients with cancer, whose tumors were interrogated for HER2 protein expression with or without amplification in a single-lab setting. To our knowledge, this is the largest database that allows examination of HER2 across diverse malignancies. Overall, 2.7 % (1014/37,992) of all tumors tested demonstrated HER2 positivity determined by immunohistochemistry (IHC), and most types of cancer had a subset of patients, albeit often small, who demonstrated HER2 positivity determined by either *in situ* hybridization (ISH) and/or IHC. These data form the foundation for possible studies that assess HER2 therapy in a pan-cancer fashion.

## Materials and methods

### Tissue samples

Solid tumor specimens submitted to a commercial molecular profiling laboratory (Caris Life Sciences, Phoenix, Arizona; a CLIA, CAP, NYSDOH and ISO certified laboratory) were initially evaluated for this retrospective analysis of HER2. The pathologic diagnosis was obtained from the pathology report provided by the outside lab and was further reviewed and verified by board-certified pathologists at Caris. Tissue requirements and detailed processing methods in collection locations can be found in the Supplemental Methods. Multi-platform profiling included immunohistochemistry and *in situ* hybridization either by fluorescent *in situ* hybridization (FISH) or chromogenic *in situ* hybridization (CISH). This investigation was performed in accordance with UC San Diego IRB guidelines.

### Immunohistochemistry

IHC analysis was performed on formalin-fixed, paraffin-embedded tissue utilizing the commercially available antibody PATHWAY anti-HER2 (4B5) rabbit monoclonal primary antibody (Ventana Medical Systems). All IHCs were performed using commercially available detection kits and automated membranous staining techniques (Benchmark XT, Ventana, USA). HER2 scoring was reported per American Society of Clinical Oncology/College of American Pathologists (ASCO/CAP) guidelines published in 2007 [14] and updated in 2013 [19, 20]. For the purpose of our analysis, an IHC test was considered positive (IHC+) when IHC3+ was obtained above the guidelines defined thresholds; an IHC test was considered negative (IHC-) when IHC 2+ (equivocal), IHC 1+, or IHC 0 was obtained.

### HER2 *in situ* hybridization

Among the 37,992 samples analyzed by IHC, 21,642 samples were also examined with ISH. FISH was used for evaluation of the *HER2* amplification status. *HER2/CEP17* ratio higher than 2.2 was considered amplified [14] (ISH+), and HER2/CEP17 ratio between 1.8 and 2.2 (equivocal) in FISH or HER2/CEP17 ratio <1.8 in FISH was considered non-amplified (ISH-). *HER2* amplification was also evaluated by CISH (INFORM HER2 dual ISH DNA probe cocktail, Ventana). Consistent with the CISH package insert, *HER2/CEP17* ratio higher than 2.0 was considered amplified (ISH+); HER2/CEP17 ratio <2.0 in CISH was considered non-amplified (ISH-).

#### HER2 fluorescent *in situ* hybridization

FISH was performed with a probe specific for *HER2* (17q11.2-q12 region) and a probe for the pericentromeric region of chromosome 17 (Abbott Molecular/Vysis). Interphase nuclei were examined and the ratio of HER2 signals to chromosome 17 centromere signals were evaluated to indicate amplification status of this gene. The HER2 Pathvysion probe has been approved by the US Food and Drug Administration for selection of patients for trastuzumab and pertuzumab therapy.

#### HER2 chromogenic *in situ* hybridization

CISH was performed by using Ventana Medical Systems, Inc.’s (Ventana) INFORM HER2 Dual ISH DNA Probe Cocktail as intended to determine HER2 gene status by enumeration of the ratio of the HER2 gene to chromosome 17. The HER2 and chromosome 17 probes are detected using two color ISH in formalin-fixed, paraffin-embedded human cancer tissue specimens following staining on VENTANA BenchMark XT automated slide stainer, and visualized by light microscopy. The INFORM HER2 Dual ISH DNA Probe Cocktail has been approved by the US Food and Drug Administration for selection of patients to HER2 targeted therapies in breast cancer.

### Statistical methods

Descriptive statistics were used. For Table 2, the concordance between the IHC and ISH tests was calculated by dividing the number of samples that had concordant results for both IHC and ISH tests (IHC+ and ISH+; or IHC- and ISH-) by the total number of samples within each malignancy types. JMPv10.0 (SAS Institute Inc., Cary, NC) was utilized.

## Results

### HER2 protein expression in various malignancies

**Fig. 1:**
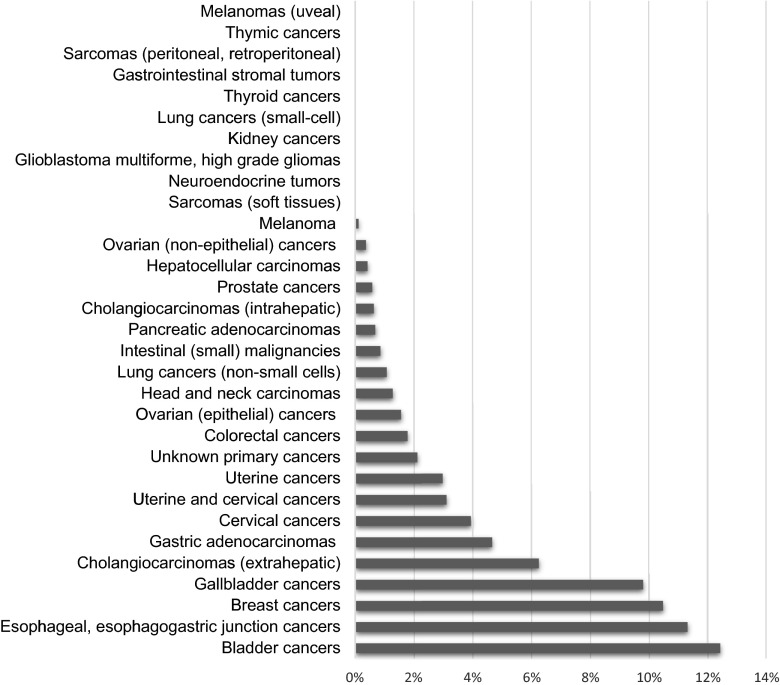
HER2 positivity across cancers: analysis of 37,992 samples by IHC. IHC 3+ was considered HER2 IHC positive

As shown in Table 1 and Fig. 1, HER2 protein 3+ expression by IHC was demonstrated in a subset of virtually all examined carcinomas derived from epithelial origin. The frequency ranged from 0.4 % in hepatocellular carcinoma to 12.4 % in bladder cancer. Table 3 gives examples of positivity rates found in the literature. Interestingly, HER2 protein 3+ expression was very rare, often non-existent, in malignancies of non-epithelial origin. In 965 melanoma samples, only one showed HER2 3+ expression. In 1,211 sarcomas (soft tissues) and 1,136 neuroendocrine tumors, none exhibited 3+ HER2 protein expression. No HER2 3+ expression was detected in gastrointestinal stromal tumors (GIST), small cell lung cancers (SCLC), kidney cancers, and glioblastomas.

**Table 1:** HER2 positivity by IHC in diverse cancers (*N* = 37,992)

Malignancy type	HER2 positive (IHC 3+) samples (N)	Total no. of samples	Percentage of HER2 positivity (%)
• *N* > 50 in each cancer type	*N* = 1013	*N* = 37,864	2.7
Bladder cancers	59	475	12.4
Breast cancers	388	3706	10.5
Cervical cancers	23	585	3.9
Cholangiocarcinomas (extrahepatic)	5	80	6.3
Cholangiocarcinomas (intrahepatic)	2	321	0.6
Colorectal cancers	80	4507	1.8
Esophageal, esophagogastric junction cancers	71	628	11.3
Gallbladder cancers	19	194	9.8
Gastric adenocarcinomas	27	580	4.7
Gastrointestinal stromal tumors	0	143	0.0
Glioblastoma multiforme, high grade gliomas	0	763	0.0
Head and neck carcinomas	7	552	1.3
Hepatocellular carcinomas	1	245	0.4
Intestinal (small) malignancies	2	235	0.9
Kidney cancers	0	531	0.0
Lung cancers (non-small cells)	49	4609	1.1
Lung cancers (small-cells)	0	322	0.0
Melanomas	1	965	0.1
Melanomas (uveal)	0	54	0.0
Neuroendocrine tumors	0	1136	0.0
Ovarian (epithelial) cancers	122	7854	1.6
Ovarian (non-epithelial) cancers	1	279	0.4
Pancreatic adenocarcinomas	14	2072	0.7
Prostate cancers	2	350	0.6
Sarcomas (peritoneal, retroperitoneal)	0	106	0.0
Sarcomas (soft tissues)	0	1211	0.0
Thymic cancers	0	90	0.0
Thyroid cancers	0	158	0.0
Unknown primary cancers	29	1376	2.1
Uterine cancers	111	3737	3.0
• N < 50 in each cancer type	*N* = 1	*N* = 128	0.8
Gliomas (low-grade)	0	41	0.0
Oligodendrogliomas	0	18	0.0
Penile cancers	0	10	0.0
Pituitary cancers	0	6	0.0
Solitary fibrous tumors	0	12	0.0
Testicular cancers	1	41	2.4
Overall	*N* = 1014	*N* = 37,992	2.7

### HER2 positivity determined by IHC and ISH in various malignancies

HER2 protein expression determined by IHC and HER2 gene amplification determined by ISH were examined in a total of 21,642 samples. Consistent with HER2 protein expression pattern, HER2 gene amplification was predominantly detected in malignancies derived from epithelial origin and very rarely found in cancers derived from other tissue origins; HER2 amplification was seen in only one out of 60 retroperitoneal/peritoneal sarcomas, four out of 237 kidney cancers, and none in GISTs (0/59), small cell lung cancers (0/108), or melanomas (0/388). A total of 705 samples were found to be HER2 positive by IHC; 638 (90.5 %) of them were also positive by ISH testing. Among breast cancer cases, 286 samples tested IHC+; 266 of them (93 %) were also positive by ISH. The overall concordance rate (IHC+ and ISH+; or IHC- and ISH-) between the two tests was 96.3 % (Table 2).

**Table 2:** Concordance of HER2 positivity determined by both IHC and ISH in diverse cancers (*N* = 21,642)^a^

Malignancy type	No. of samples tested	Concordance IHC/ISH (*N*, %)
Bladder cancers	323	291 (90.1 %)
Breast cancers	2886	2754 (95.4 %)
Cervical cancers	303	294 (97.03 %)
Cholangiocarcinomas (extrahepatic)	34	28 (82.4 %)
Cholangiocarcinomas (intrahepatic)	122	120 (98.4 %)
Colorectal cancers	1636	1588 (97.1 %)
Esophageal, esophagogastric junction cancers	575	522 (90.8 %)
Gallbladder cancers	75	69 (92.0 %)
Gastric adenocarcinomas	494	478 (96.8 %)
Gastrointestinal stromal tumors	59	59 (100 %)
Glioblastoma multiforme, high grade gliomas	424	423 (99.8 %)
Gliomas (low grade)	33	33 (100 %)
Head and neck carcinomas	257	252 (98.1 %)
Hepatocellular carcinomas	125	121 (96.8 %)
Intestinal (small) malignancies	122	114 (93.4 %)
Kidney cancers	237	233 (98.3 %)
Lung cancers (non small cells)	2188	2109 (96.4 %)
Lung cancers (small cells)	108	108 (100 %)
Melanomas	388	388 (100 %)
Melanomas (uveal)	16	16 (100 %)
Neuroendocrine tumors	399	399 (100 %)
Oligodendrogliomas	3	3 (100 %)
Ovarian (epithelial) cancers	6347	6132 (96.6 %)
Ovarian (non-epithelial) cancers	270	269 (99.6 %)
Pancreatic adenocarcinomas	844	805 (95.4 %)
Penile cancers	5	5 (100 %)
Pituitary cancers	3	3 (100 %)
Prostate cancers	154	149 (96.8 %)
Sarcomas (peritoneal, retroperitoneal)	60	59 (98.3 %)
Sarcomas (soft tissues)	432	430 (99.5 %)
Solitary fibrous tumors	2	2 (100 %)
Testicular cancers	18	16 (88.9 %)
Thymic cancers	26	26 (100 %)
Thyroid cancers	71	71 (100 %)
Unknown primary cancers	635	611 (96.2 %)
Uterine cancers	1968	1872 (95.1 %)
Overall	21,642	20,852 (96.3 %)

^a^11,670 patients had IHC and FISH; 9972 patient, IHC and CISH. IHC test was considered positive (IHC+) when IHC 3+ was obtained. ISH test was considered positive (ISH+) when the HER2/CEP17 ratio was >2.2 (by FISH) or 2.0 (by CISH) [14]. Concordance was defined as both tests being positive or negative (IHC+ and ISH+; or IHC- and ISH-).

## Discussion

Our recent review of the literature that examined HER2 amplification/overexpression in a variety of cancers outside of breast and stomach [13] found that almost all studies focused on HER2 status in one type of malignancy, making it difficult to compare the rate of HER2 positivity across studies and tumor types. Herein, we have analyzed 37,992 patients with cancer whose tumors were interrogated for HER2 protein expression in a single, accredited laboratory setting. We report that 2.7 % of all cancer samples demonstrated HER2 overexpression (3+ on IHC) (Table 1). To our knowledge, this is the largest database that interrogated HER2 across diverse malignancies. These observations provide a consistent comparison of HER2 status between individual tumor types that could be used to inform future trials of anti-HER2 therapy outside of breast and stomach cancer. For example, the incidence of HER2 overexpression by IHC in bladder cancers (12.4 %) is even higher than that found in breast cancer (10.5 %) (Fig. 1), suggesting that a trial of anti-HER2 agents may be warranted in patients with advanced bladder cancer. HER2 overexpression by IHC was also substantial in gallbladder cancers (9.8 %). When reviewing the literature, it was noticeable that studies demonstrated variability in rates of HER2 overexpression (Table 3), making our results difficult to compare with other published series. This variability can be explained by the differing criteria used for the evaluation of HER2 positivity in the studies found in the literature, due in part by the lack of standardized methodology for HER2 detection outside of gastric and breast cancers [13]. Of interest, we report that approximately 10.5 % of breast cancer patients are HER2 positive by IHC, which appears to be in the lower range when compared to published reports (11–25 %, Table 3). Varga et al. [26] investigated HER2 positivity in more than 7,000 patients with breast cancers over a 12-year period and showed a drop in the expression rate (probably due to the modified ASCO criteria in 2007), which went from 22 to 12 % over the years. The decrease in HER2 positivity rate can also potentially be explained by the introduction of mammography screening with improved detection of early breast cancers, as screen detection resulted in a shift to a different patient population with less HER2 positive cases in early breast cancer and with younger age at diagnosis [39, 40]. Lastly, the lower percentage of HER2 positivity rate in our study may be due to a higher proportion of triple-negative breast cancer specimens sent to the testing laboratory (35.8 % (Caris, data on file) as compared to 15–20 % of the general breast cancer population [19, 41]). Due to the aggressive nature of triple-negative breast cancer, a higher percentage may be evaluated for the molecular profiling that generally is performed with HER2 testing in the laboratory. One interesting pattern of HER2 positivity became apparent with direct comparison of HER2 status across different tumor types: overexpressed HER2 is predominantly found in malignancies of epithelial origin. For cancers derived from mesenchyme, neuroendocrine tissue, central nervous system, and kidney, HER2 expression and HER2 gene amplification are negligible (Fig. 1).

**Table 3:** Examples of HER2 positivity frequencies by IHC

Malignancy type	Percentage of HER2 positivity in literature [13]	References
Bladder cancers	8–70	[21–25]
Breast cancers	11–25	[26, 27]
Cervical cancers	2.8	[28]
Cholangiocarcinomas	9 (extrahepatic) ∼1 (intrahepatic)	[29]
Colorectal cancers	1.6–5	[30–32]
Esophageal, esophagogastric junction cancers	12–14	[33–35]
Gallbladder cancers	12.8	[36]
Gastric adenocarcinomas	7–34	[37, 38]

HER2 gene amplification is the major mechanism driving HER2 overexpression in breast cancer [39]. It has been noted before that HER2 protein may not be consistently analyzed in formalin-fixed tissues because of variations in methods and duration of fixation, in comparison to ISH analysis that is less dependent on tissue fixation methods [14, 39, 40]. Alternatively, it is plausible that there are transcriptional or translational mechanisms that could attenuate HER2 expression in some cases, even in the presence of amplification. The overall (positive and negative) concordance rate between methodologies was about 96 % in our cohort, which is in accordance with the American Society of Clinical Oncology/College of American Pathologists guideline that any two diagnostic companion tests should establish a concordance rate of >95 % for positive and negative assay values [14]. Further, 90 % of all samples and 93 % of breast cancer cases that were tested HER2 positive by IHC, were also found positive by ISH. This percentage stands in the higher range of what has been published. For example, the positivity concordance rates (IHC+ tests also found positive by FISH) reported by different groups ranged from 69 to 98 %, with most of them being around 85 % [26, 41–43].

There are several limitations to our study. The current pool of samples cover a wide range of malignancies but there may be selection bias of the specimens, as patients/physicians may have elected to submit tissue when there were fewer standard therapeutic options left, and often for advanced and/or more difficult to treat cancers (refractory and recurrent). Also, the samples came from diverse institutes and practices, and differences in processing and storage could still have affected samples, even if the HER2 analysis itself was performed by a single organization. Finally, the current study addressed HER2 overexpression. It has recently been shown that HER2 can demonstrate mutations in about 1.8 % of diverse cancers (∼7300 solid tumor specimens tested) and that rearrangements can be seen, albeit very rarely (∼0.02 % of patients) [44]. In summary, we have examined 37,992 samples for HER2 expression (with 21,642 of them also examined for HER2 amplification) and profiled HER2 status across different tumor types. Overall, 2.7 % of all cancers tested were IHC 3+ positive. High levels of HER2 were seen in a subset of patients with most epithelial malignancies examined. For non-epithelial cancers such as melanoma, GIST, small cell lung cancers, and glioblastomas, almost no HER2 overexpression was discerned. Some tumors such as bladder, gallbladder, and cholangiocarcinomas showed significant rates of HER2 positivity (greater than 5 %), and testicular, ovarian, uterine, cervical, head and neck, non-small cell lung, and colorectal cancer as well as tumors of unknown origin all showed small but not negligible rates of positivity. Of additional interest, a small percentage of tumors may also have HER2 mutations that are amenable to targeted HER2 agents, and these patients would not be expected to have high expression levels of HER2 [13, 15–18]. An early histology-independent trial using lapatinib monotherapy for HER2-expressing tumors faced logistical challenges and showed only modest activity [45]. However, in the last several years, there has been increasing experience with target-driven trials and overcoming their logistical challenges [46–48]. Our data presented herein, as well as the advent of numerous, potent small molecule inhibitors, antibodies and other agents that target HER2, suggest that it may be worthwhile to enrich clinical trials using HER2-targeting agents with patients that have HER2-positive tumors other than breast and gastric.

## Electronic supplementary material

Below is the link to the electronic supplementary material.

ESM 1 (DOC 37 kb)
