# Supplementary material for: HER2 expression status in diverse cancers: review of results from 37,992 patients
Source: Cancer Metastasis Rev. 2015 Feb 25;34(1):157–64. doi: 10.1007/s10555-015-9552-6 (PMC4368842; doi:10.1007/s10555-015-9552-6)
Supplement: Supplementary file 1 — (DOC 37 kb) [file 10555_2015_9552_MOESM1_ESM.doc]

**SUPPLEMENTAL METHODS**

**Processing of tissue samples and requirements:**

**A. Formalin Fixed Paraffin Embedded (FFPE) Samples:**

1) Fixed Tissue: One (1) tumor-containing formalin fixed paraffin embedded block (FFPE) from most recent surgery or biopsy. Successive four (4) micron sections will be created from the block until sufficient material for the testing orders is obtained. For the molecular analysis, tumor cells will be excised by micro-dissection until a total area of at least 50mm^2^ is obtained.

2) Core Needle Biopsy: Four to six (4-6) biopsies formalin fixed paraffin embedded–18 gauge needle preferred–

3) Fine Needle Aspirate (FNA): One (1) formalin fixed paraffin embedded block containing sufficient tumor.

4) Unstained Slides: Unstained, positively charged, unbaked slides from one single, tumor-containing formalin fixed paraffin embedded block; 4 micron sections (MI Profile™ - 55 slides; Next-Generation Sequencing only - 15 slides). Note: At least a 5mm x 5mm section of tissue per slide is required. For small biopsies (tissue area < 5 mm x 5 mm) please cut two sections per slide for at least one half of the slides to ensure sufficient material for molecular assays.

5) Malignant Fluid: One (1) formalin fixed paraffin embedded cell block containing sufficient tumor.

**B. Formalin Samples**

1) Fresh Tissue: Two (2) or more samples with a minimum thickness of ~3mm (height, width, length) and submit in 10% neutral buffered formalin.

2) Core Needle Biopsy: Four to six (4-6) biopsies–18 gauge needle preferred–

3) Bone/Bone Metastasis: Two (2) or more samples with minimum thickness of 3mm (height, width, length) and submit in 10% neutral buffered formalin (not decalcified).
